# Challenges in Additive Manufacturing of Space Parts: Powder Feedstock Cross-Contamination and Its Impact on End Products

**DOI:** 10.3390/ma10050522

**Published:** 2017-05-12

**Authors:** Ana D. Brandão, Romain Gerard, Johannes Gumpinger, Stefano Beretta, Advenit Makaya, Laurent Pambaguian, Tommaso Ghidini

**Affiliations:** 1 ESTEC, European Space Agency, 2200 AG Noordwijk, The Netherlands; ana.brandao@esa.int (A.D.B.); romain.l.gerard@gmail.com (R.G.); advenit.makaya@esa.int (A.M.); laurent.pambaguian@esa.int (L.P.); tommaso.ghidini@esa.int (T.G.); 2 Politecnico di Milano, Via La Masa 1, 20156 Milan, Italy; stefano.beretta@polimi.it

**Keywords:** Additive Manufacturing, tensile properties, microstructure, cross contamination, space applications, Ti-6Al-4V

## Abstract

This work studies the tensile properties of Ti-6Al-4V samples produced by laser powder bed based Additive Manufacturing (AM), for different build orientations. The results showed high scattering of the yield and tensile strength and low fracture elongation. The subsequent fractographic investigation revealed the presence of tungsten particles on the fracture surface. Hence, its detection and impact on tensile properties of AM Ti-6Al-4V were investigated. X-ray Computed Tomography (X-ray CT) scanning indicated that these inclusions were evenly distributed throughout the samples, however the inclusions area was shown to be larger in the load-bearing plane for the vertical specimens. A microstructural study proved that the mostly spherical tungsten particles were embedded in the fully martensitic Ti-6Al-4V AM material. The particle size distribution, the flowability and the morphology of the powder feedstock were investigated and appeared to be in line with observations from other studies. X-ray CT scanning of the powder however made the high density particles visible, where various techniques, commonly used in the certification of powder feedstock, failed to detect the contaminant. As the detection of cross contamination in the powder feedstock proves to be challenging, the use of only one type of powder per AM equipment is recommended for critical applications such as Space parts.

## 1. Introduction

Additive Manufacturing (AM), or 3D printing includes a large family of processes having a common basic principle: the continuous, local addition of material to obtain fit-for-purpose hardware [1,2]. This is opposed to classical machining processes, where material is cut away from large blocks of so-called “semi-finished products”. AM allows an increase in design freedom and, hence, is seen as one key enabling technology for high end manufacturing, including Space applications.

In fact, the manufacture of parts for Space using AM has already started, as described in [3,4]. However, current applications mainly focus on secondary structures or other non-critical applications, manufactured on earth. For future missions, many more components are envisioned to be manufactured using AM, including primary structures or other mission-critical parts, and even the production of these parts in orbit. The Space domain underlines the vast potential of AM regarding costs savings and increased performance.

In this context, and from the acquired experience, it appears essential for AM based Space hardware to establish an “end to end manufacturing process”. This should be initiated as early as the initial design phase, leading to the material supply, the manufacturing itself, post processing and qualification. Following this approach, it is necessary to ensure consistent mechanical and physical properties to meet stringent reliability requirements for components used for Space applications, as for other highly demanding uses [5,6,7].

One of excellent candidate AM materials for high end applications is the Titanium alloy Ti-6Al-4V, due to its high strength, low density and high corrosion resistance [6,8]. The maturity of the AM processing of Ti-6Al-4V is an additional rationale to use it. As such, several authors have studied this alloy, namely its microstructure [8,9], tensile properties [10,11] and fatigue behaviour [5,12].

In addition, current studies show that significant variations in mechanical and physical properties may occur between finished AM products. The main drivers for these differences are not only in the processing parameters—such as the laser power or the scan speed [6,8,13,14]—but also in a variation or degradation of the feedstock [15,16].

Considering the latter, there is an increasing interest from the AM industry in developing strategies to control the properties of the powders [17,18,19], specifically their chemical composition. The alterations to the nominal chemical composition of the powder alloys used for AM may originate from different sources, e.g., raw material production stage, storage conditions, cleanliness requirements and cross-contamination from different feedstock. In particular, the oxygen content on the powder is commonly measured along these stages, as has been reported, to affect the tensile properties of Ti-6Al-4V, where its increase was found to improve the tensile strength and impair the elongation to failure [15,17].

On the other hand, the impact of powder feedstock cross-contamination on the mechanical properties of AM parts has not been extensively studied. Although it is commonly accepted [15,17,19] that cross contamination may influence the properties of the final products, a direct relationship between the presence of contamination and the mechanical behaviour of the final part has not yet been established, to the best of our knowledge.

This work reports the findings of high density inclusions in specimens produced via AM during the investigation of Ti-6Al-4V tensile properties. The impact of these inclusions on the mechanical properties of the Ti-6Al-4V samples is assessed. In addition, the tensile properties and the microstructure of Ti-6Al-4V are characterised against the build direction of the parts. Furthermore, the study intends to identify the root cause of the contamination and to evaluate different approaches to identify contaminating particles in AM feedstock.

## 2. Materials and Methods

**Figure 1:**
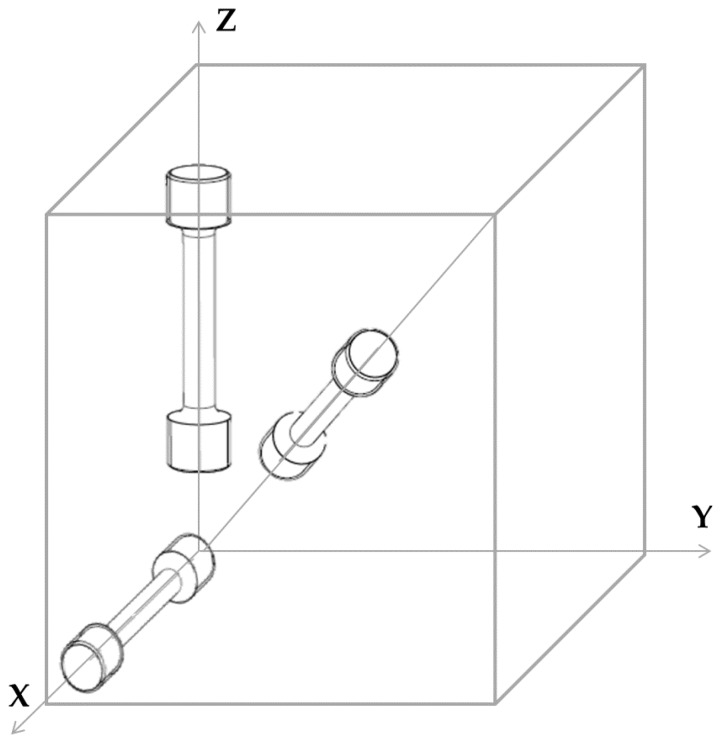
Horizontal, vertical, and inclined orientations of static tensile specimens: **Z** is the building direction, and **X** is the recoating direction.

The Ti-6Al-4V samples were manufactured on a powder bed based, selective laser melting (SLM) Additive Manufacturing machine, with an energy density of 59 J/mm³ at layer thickness of 30 µm. The manufacturing was followed by stress relief annealing at 670 °C with a dwell time of 5 h in vacuum. A pre-alloyed Ti-6Al-4V powder produced by inert gas atomization was used as the starting material. A ceramic jet blasting surface finishing technique was applied to the AM specimens, to remove adhering particles after the build job and to reduce the surface roughness. Five specimens in each of the horizontal (**X**), vertical (**Z**), and 45° (**111**) inclined orientations were manufactured, as depicted in Figure 1. The **Z** axis is the building direction, whereas powder recoating was done in **X** direction.

**Figure 2:**
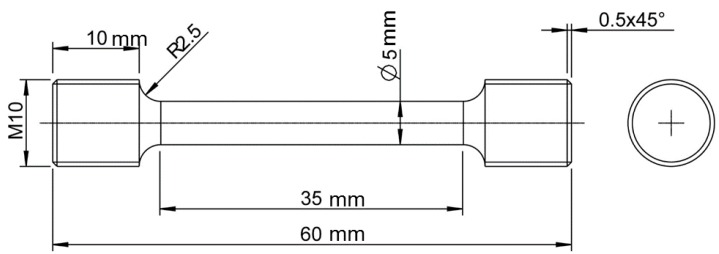
Geometry of static tensile specimens [20].

Following the testing and characterization of this first set of specimens (Batch 1), a second batch of tensile specimens was requested from the manufacturer, to serve as a control group (Batch 2). As for the Batch 1, five specimens in each of the horizontal, vertical, and 45° inclined orientations were manufactured. The parts from Batch 2 were manufactured with the same parameters and geometrically described in Figure 1 and Figure 2, using a different powder batch. Tensile testing, fracture surface analysis and X-ray Computed Tomography (X-ray CT) were performed on the second batch of specimens, using the same methods as for the first set of specimens, as described above.

### 2.1. Tensile Properties

The threads of the tensile specimens were machined according to ASTM E8M [20], the reduced section of 5 mm was not machined, see Figure 2. The tensile testing was carried out at room temperature on a Zwick/Roell Z100, according to ASTM E8M [20] using a 100 kN load cell. Fourteen specimens were tested: five specimens in the **X** orientation, five in the **111** orientation and four specimens in the **Z** orientation (refer to Figure 1). The specimens were tested with a crosshead speed control method, with an applied speed of 0.0114 mm/mm/min to determine the Young’s modulus and yield strength, until a strain value of 0.02. In the following step, the speed was increased to 0.015 mm/mm/min to calculate the tensile strength. The strain measurement was performed using the optical extensometer Aramis 3D system from GOM GmbH.

The fracture surface was analysed using Scanning Electron Microscopy (SEM), in a Zeiss LEO microscope, coupled with Energy Dispersive X-ray Spectroscopy (EDX).

### 2.2. Defect Analysis

**Figure 3:**
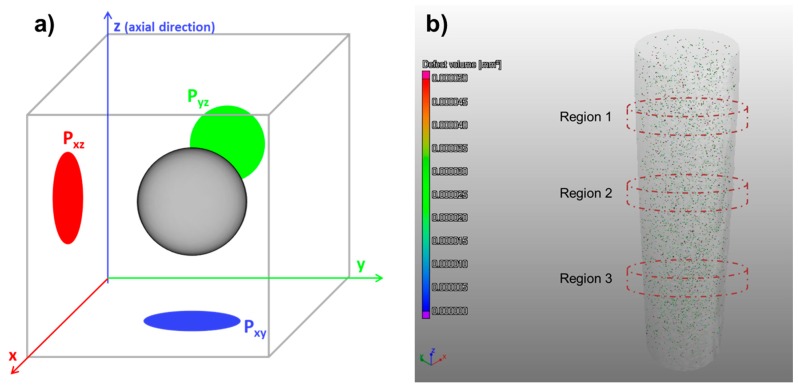
Schematic representation of: (**a**) the projections on the P_xy_, P_yz_, P_xz_ planes of the defect detected via X-ray CT (adapted from [23]); and (**b**) the regions of analysis defined for each tensile sample from Batch 1 built in **X** and **Z** directions.

The defect population of the tensile specimens, namely voids and inclusions, was characterised via X-ray CT. One specimen per build direction (**X**, **111**, **Z**) was scanned. A Phoenix v|tome|x m 300 kV (General Electric Sensing & Inspection Technologies GmbH, Wunstort, Germany) was used, at a gun voltage of 220 kV, a current of 50 µA, using 1000 ms per frame and acquiring 1500 images over the complete rotation. The voxel size was 29 µm for the horizontal sample and 25 µm for the vertical and 45° inclined samples. A detailed scan was performed on a selected region of one sample per horizontal (**X**) and vertical (**Z**) directions in order to study the dependence of defect location with build orientation. These samples were scanned with a voxel size of 8 µm. The results were analysed with the software VG Studio MAX 2.2.1 (Volume Graphics GmbH, Heidelberg, Germany). One of the parameters given by this software is the area of the projection of each defect (voids, inclusions) along the coordinate planes (P_xy_, P_yz_, P_xz_). These are schematically represented in Figure 3a), where the defect is depicted as a grey sphere and its projections, P_xy_, P_yz_, P_xz_, are portrayed as the geometrical shapes in blue, green, and red, respectively. P_xy_ represents the plane perpendicular to the axial direction of the tensile testing.

The project areas were analysed for three different, disc-shaped regions of 500 µm height in each sample, as exemplified in Figure 3b), ensuring the same volume of analysis. This method is comparable to the conventional assessment of porosity through the preparation of microsections. However, the use of X-ray CT allows the observation of a larger number of defects per region of analysis, as well as the assessment of the volumetric (three dimensions) characteristics of the defects [21,22].

### 2.3. Microstructure Characterization

Microstructural characterization of the tensile specimens was performed by optical microscopy using Leica DMI 5000 M and by SEM. A horizontal and a vertical plane from a **Z** tensile specimen were polished on a Struers TegraPol-35. The samples were polished with a final sand paper grit size of 1200 grit and subsequently with diamond suspension from 6 µm to 1 µm sizes. The microsections were then etched with a Ti ASTM 186 chemical agent for 30 s.

The Vickers Hardness (HV) was measured on the microsections of tensile specimens produced with both powder batches, on one horizontal (**X** orientation) sample per batch and one vertical (**Z** orientation) sample per batch, i.e., on a total of four samples. HV was measured using an MHT-10 Microhardness tester (Anton Paar GmbH, Graz, Austria), using a load of 200 gf with a 30 s hold time, and measuring with a 50× optical magnification.

### 2.4. Powder Investigation

The flow properties of the Ti-6Al-4V powder feedstock were determined with a Hall Flowmeter according to ASTM B213 [24], using 50 g samples. In addition, the particle size distribution of the powder was measured on a RETSCH Horiba LA 950 V2 laser light scattering device (Retsch Technology GmbH, Haan, Germany).

Determination of the phases in the powder and AM samples was performed by X-ray diffraction (XRD), using a Bruker D8 Discover diffractometer (Cu Kα radiation, λ = 1.5406 Å), (20°–100° 2θ, step 0.06, 6 s/step). Unit-cell parameters were determined from the diffraction data using the integrated DIFFRAC.EVA v3.0 software (Bruker, Karlsruhe, Germany).

In addition, a sample of the powder feedstock was analysed with the X-ray CT. The powder was enclosed in a polyethylene vial and scanned with a resolution of 6.7 µm per voxel, in order to assess the potential differences in density within the powder sample.

The chemical composition was investigated by the following techniques for the powder feedstock from the first batch and the bulk AM samples from the first and second batches. Inductively Coupled Plasma Optical Emission Spectrometry (ICP OES) was used to determine the Ti, Al, V and W contents. Carrier Gas Hot Extraction (CGHE) was used for the H, O, and N contents. The C content was determined by infrared absorption method, after combustion in an induction furnace. Flame Atomic Absorption Spectroscopy (AAS) was used to determine the Fe content.

## 3. Results and Discussion

### 3.1. Mechanical Properties

The tensile testing results of the first Ti-6Al-4V batch, in the stress relief annealed condition, are shown in Table 1. The data shown in the table correspond to arithmetic averages for each specimen orientation: i.e., average of five specimens in the **X** orientation, average of five specimens in the **111** orientation and average of four specimens in the **Z** orientation.

The samples in all orientations were above the threshold of 825 MPa for the yield strength and 895 MPa for the tensile strength for Ti-6Al-4V specimens according to ASTM F2924 [25] for class F components in the stress relief annealed condition. In the case of fracture elongation, the values for both **X** and **111** orientations meet the requirements of 6% specified in [25] for class F components. However the samples built in the **Z** orientation present fracture elongation values significantly lower than in the other orientations and are below the threshold given in ASTM F2924 [25]. Kobryn et al. [26] and Qui et al. [27] reported a similar anisotropy for the fracture elongation values of specimens built in various orientations. Both authors suggest that the impaired elongation values for the samples built in the **Z** orientation was justified by the presence of lack-of-fusion-related porosity. This defect tends to form along the interfaces between successive build layers, perpendicular to the vertical build direction, resulting in the reduction of load-bearing area during the tensile testing of these specimens. In addition, Kobryn et al. [26] and Vrancken et al. [14] reported fracture elongation values lower than the ASTM F2924 [25] requirement for class F components, for specimens in the stress relief annealed condition. The tendency of a decreasing fracture elongation with increasing cooling rate for Ti-6Al-4V is described in [28]. The authors also report a significantly lower ductility for acicular microstructure as present within this work, when compared to bi-modal microstructure [28].

**Figure 4:**
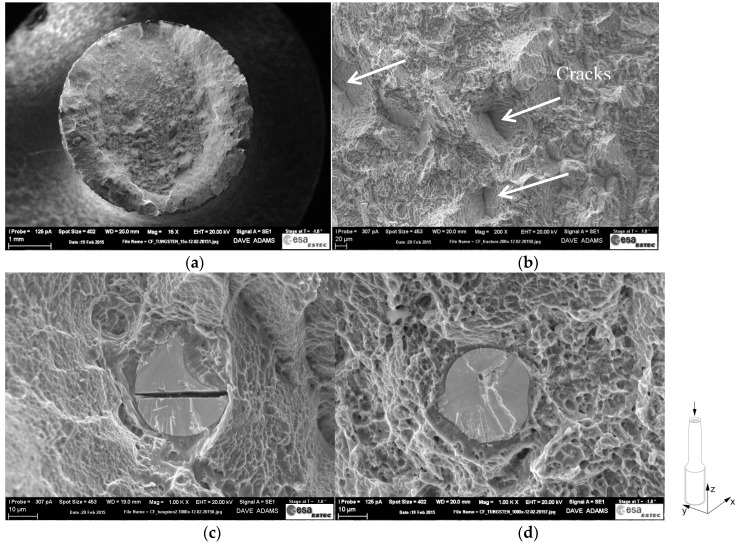
SEM images of the fracture surface of a tensile specimen in the **Z** orientation: (**a**) overview of the fracture surface; (**b**) close-up view of cracks; (**c**) close-up view of cracked inclusion with a brittle appearance; (**d**) close-up view of inclusion with a brittle appearance.

To better understand this anisotropy in the fracture elongation the fracture surface of a selected specimen, built in the vertical orientation was investigated. The main features of the fracture surface observed are reported in Figure 4. The general appearance of the investigated specimen features the classic cup-and-cone tensile fracture surface with an inner fibrous zone, outer shear lips, and dimples. Dimples were observed on the shear lips as well as on the fibrous zone, as shown in Figure 4a. The fibrous zone also exhibited cracks, as depicted in Figure 4b, which is in accordance with observations reported in [29] for Ti-6Al-4V specimens produced by laser powder bed based AM. Embedded particles with brittle appearance are shown in Figure 4c,d. The EDX analysis detected a tungsten content of more than 90 wt % in these particles. No evidence of lack-of-fusion was found on the fracture surface. As mentioned above, lack-of-fusion is commonly observed for AM parts [9,14,27,29,30] and has a strong impact on the mechanical properties of Ti-6Al-4V AM parts. The results obtained in this work suggest that the presence of the W particles has a similar impact on the mechanical properties of the AM Ti-6Al-4V tensile specimens as the lack-of-fusion defects reported by Kobryn et al. [26] and Qui et al. [27]. The melting temperature of W is significantly higher (3422 °C [31]) than the liquidus temperature of the Ti-6Al-4V alloy (1660 °C [32]). In addition, the W is characterised as a hard material, with low ductility, in the conditions here considered [33]. Consequently, the unmelted brittle W particles are expected to act as preferred sites for crack initiation in the tensile specimens. The observation of W particles with a brittle fracture appearance in the specimens’ fracture surface, Figure 4, tends to corroborate such a mechanism. As a result, the elongation of the tensile specimens is adversely impacted by the facilitated crack propagation. The reason for the larger impact on the elongation in the **Z** orientation, compared to the **X** and **111** orientations, is still not fully explained.

**Figure 5:**
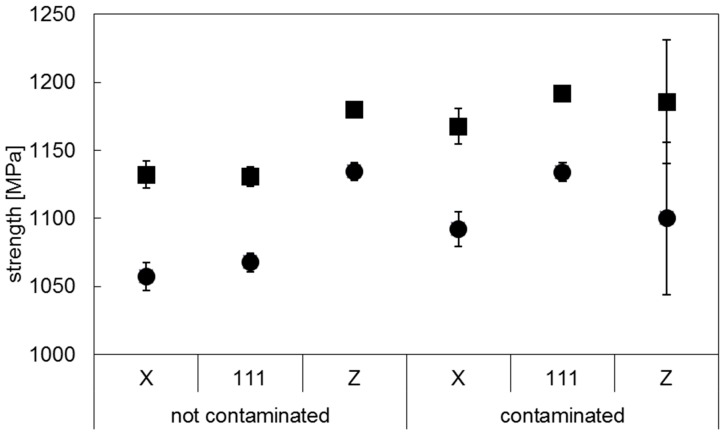
Strength values of non-contaminated (Batch 2) and contaminated (Batch 1) Ti-6Al-4V, in the stress relief annealed condition (■ tensile strength, ● yield strength).

**Figure 6:**
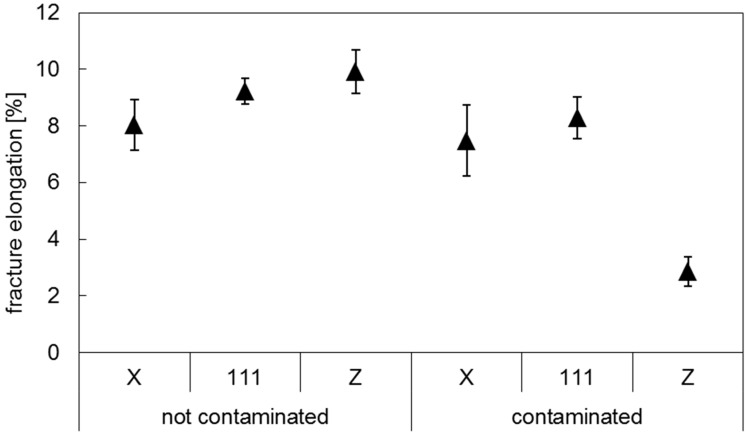
Fracture elongation of non-contaminated (Batch 2) and contaminated (Batch 1) Ti-6Al-4V, in the stress relief annealed condition.

After detecting particles on the fracture surface of the tensile specimens, it was decided to repeat the tensile tests with a second batch of specimens, as a control group (Batch 2). The second batch of AM specimens was manufactured from a different batch of Ti-6Al-4V powder feedstock. In Figure 5 and Figure 6 the results of the tensile tests of Ti-6Al-4V Batch 2 are compared to the values obtained for Batch 1. Five samples were tested in the **X** orientation, five samples in the **111** orientation and four samples in the **Z** orientation. The data are presented as arithmetic averages for each sample orientation. Error bars represent the standard deviation. Fractography performed on Batch 2 tensile samples did not exhibit any tungsten particles.

The specimens where high density inclusions were observed are referred to hereafter as “contaminated” (Batch 1), while the specimens which did not show high density inclusions are referred to as “non-contaminated” (Batch 2). The tensile testing results of the latter batch showed that the highest strength and fracture elongation values were achieved for the specimens built in **Z** orientation.

The strength values are higher for the contaminated specimens, with the exception of the yield strength of the contaminated specimens in the **Z** orientation. However, it should be noted that the strength values of the contaminated specimens in the **Z** direction show a large scattering. These results are further discussed in Section 3.2.

The results of the contaminated samples in the **Z** orientation are of particular interest as they show similar average strength values as in the other orientations, but wider scattering and drastically lower fracture elongation. The non-contaminated test specimens in the **Z** orientation show the highest elongation results with above 10%. Their contaminated counterparts show the lowest elongation at rupture with 4%.

### 3.2. Defect Distribution Analysis

**Figure 7:**
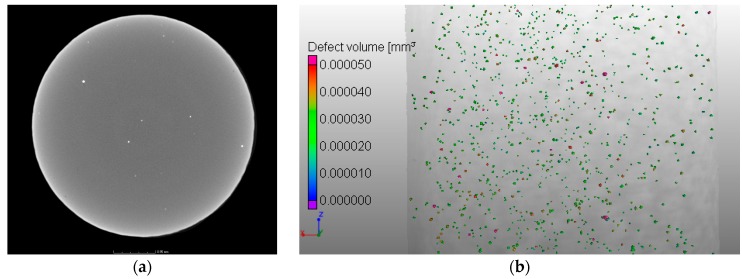
X-ray CT images of a contaminated (Batch 1) tensile sample built in **Z** direction showing (**a**) high density inclusions (white particles); and (**b**) distribution of these defects in the part, coloured according to their associated volume.

**Figure 8:**
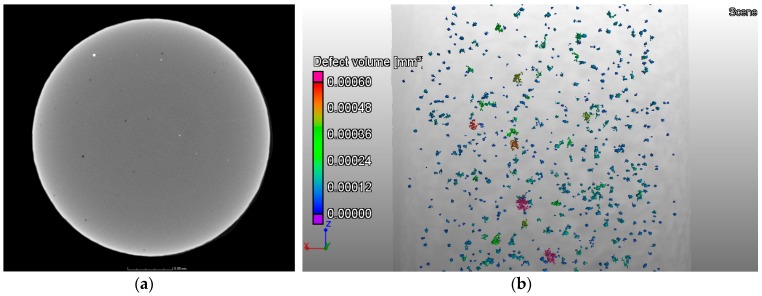
X-ray CT of a tensile sample from Batch 1 built in **Z** direction showing (**a**) the porosity (dark regions); and (**b**) distribution of these defects in the part, coloured according to their associated volume.

X-ray CT scans were performed on one sample per build direction of contaminated tensile specimens (Batch 1), showing high density inclusions in all specimens (see Figure 7) as well as porosity (see Figure 8). The fraction of high density inclusions in the analysed samples was found to be in the range of 0.03% to 0.04% by volume. The diameter of the detected high density inclusions varied from 27 µm to 140 µm for all the scanned samples from Batch 1. In the case of porosity, the volume fraction was 0.03% for the vertical specimen and close to 0.3% for horizontal and 45° inclination samples. Here the diameter ranged from 37 µm to 250 µm.

A similar characterization of the defect population of the non-contaminated samples (Batch 2) was performed using the results obtained through X-ray CT scans. The values showed that the volume fraction of defects was close to 0.2%, with the diameter varying from 40 µm to 300 µm. These results are consistent with the ones measured for the contaminated samples. The presence of high density inclusions on Batch 2 samples was considered negligible (<0.001%).

Several authors report the use of tomography techniques to study the defect population (size, density, location) in AM parts, establishing correlations with process parameters [34,35], heat treatment [21], and fracture resistance and fatigue behaviour [22]. In these investigations the analysis was performed considering the voids (pores, lack of fusion) present in the samples. This work attempts to replicate this approach in the assessment of the size distribution of the higher density particles.

**Figure 9:**
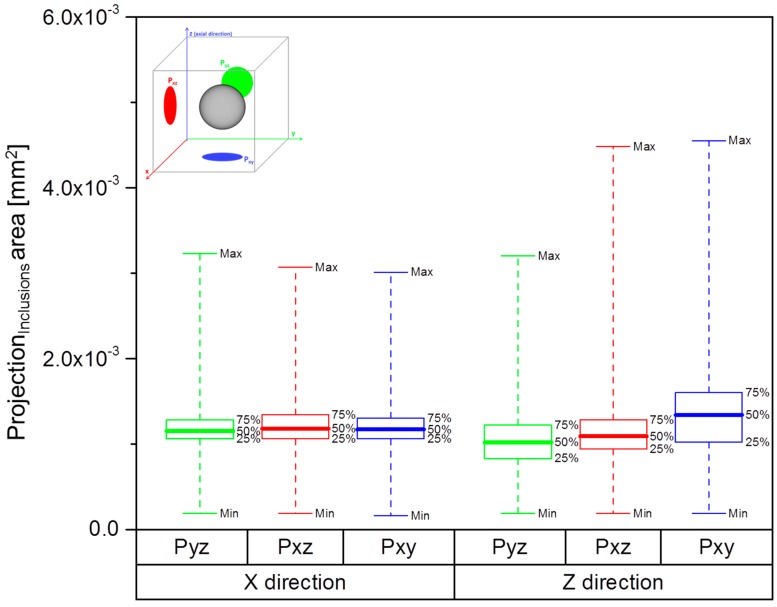
Box plot of area of the projections of the inclusions of samples built in **X** and **Z** directions, in the yz, xz and xy planes.

The projection area distribution in each plane (P_xy_, P_yz_, P_xz_) is presented in Figure 9, for samples built in **X** and **Z** directions. The data are represented as box plot, allowing a simple visualization of the projections area distributions. This method is recommended to plot population distributions that may be asymmetric and present extreme outliers [36], and it was used by Seifi et al. in a similar study [22]. The graphs in Figure 9 are defined by the minimum, first quartile (25th percentile), the median (50th percentile), third quartile (75th percentile) and the maximum. It is visible that the data of the sample built in **Z** direction show more variability, given by the larger interquartile range (IQR) between the first and the third quartiles. These spreads of the inclusions area are more evident for the projection P_xy_, perpendicular to the tensile load direction. In addition, it is seen in Table 2 that the total number of inclusions detected is larger for the sample built in **Z** direction (for same volume) and, therefore, the total area of defects is higher, when compared with the horizontal (**X** direction) sample. This difference in the total area is greater for the projections in a plane perpendicular to tensile load.

Considering this area distribution in the samples built in **Z** direction, one may infer a correlation between the mechanical properties measured, Figure 5 and Figure 6, and the presence of the higher density inclusions. In literature the influence of defects, such as porosity, on the tensile properties of metals is widely studied [37,38,39], including for Ti alloys produced by AM [26,40,41,42]. The presence of defects is commonly accepted to reduce the load-bearing area in tension and increase the stress concentration [26,37,38,39,40,41,42]. In this work it is assumed that the higher density particles produce the same effect, impairing the mechanical properties of AM Ti-6Al-4V. This effect is strongly observed for the vertical samples, as these show the presence of a larger number of inclusions, with an increased area occupied by these particles. This implies a greater reduction of the cross-sectional area in tension, as well as the creation of a network of stress concentration locations in the sample. This is supported by the observations of a brittle-like aspect of these inclusions, Figure 4. Moreover, the larger variability of the area of the inclusions in the xy plane could explain the larger scatter observed in Figure 5 for the tensile strength of the vertical samples from Batch 1 To further support these observations, a systematic study of the impact of inclusions (with well-known volume concentration) on the mechanical properties of AM parts will be performed in future work.

### 3.3. Microstructure

**Figure 10:**
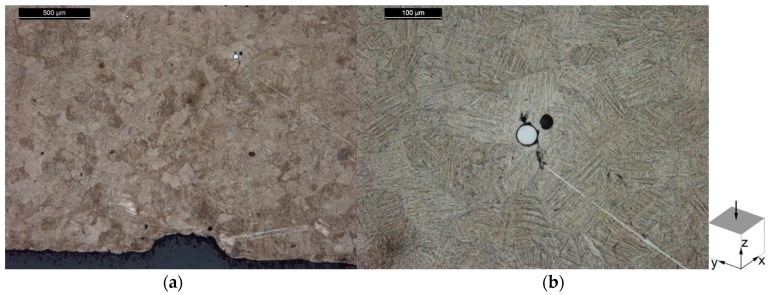
Microstructure of the horizontal plane of Batch 1 Ti-6Al-4V tensile specimen, in the stress relieve annealed condition showing: (**a**) an overview of the microstructure with an embedded particle; and (**b**) the referred particle at higher magnification.

**Figure 11:**
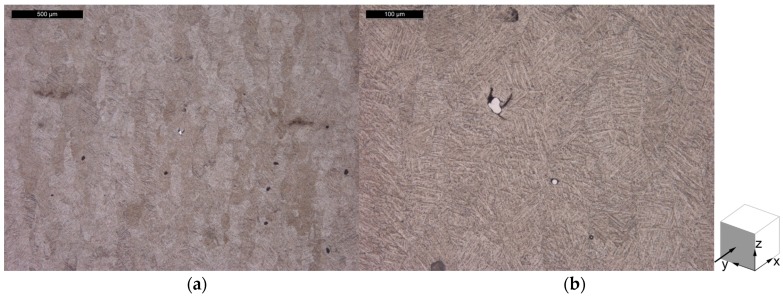
Microstructure of the vertical plane of Batch 1 Ti-6Al-4V tensile, in the stress relieve annealed condition showing: (**a**) an overview of the microstructure with an embedded particle; and (**b**) the referred particle at higher magnification.

The microstructure of the specimens from Batch 1 is shown in Figure 10 for the horizontal plane and in Figure 11 for the vertical plane. Entrapped W particles, which microstructure is not revealed by the ASTM 186 chemical etchant, are visible in these images. An isotropic microstructure is observed in the horizontal plane, as seen in Figure 10a, while the vertical plane seen in Figure 11a shows elongated grains. From this observation it was concluded that the microstructure is composed of columnar grains orientated along the build direction (perpendicular to the base plate). This characteristic microstructure has been attributed to the interaction of the laser with the powder bed, where the top layer is molten and the previous layers are heated and partly molten [43,44,45]. Heat is then conducted in the vertical build direction, leading to solidification and epitaxial growth of the grains in this direction.

The XRD study of the powder samples confirms the presence of a hexagonal closed-pack crystal structure corresponding to the martensite phase (α′). The lattice parameters recorded for the powder material are a = 2.929 ± 0.029 Å and c = 4.660 ± 0.047 Å, which are in accordance with previously reported values for Ti-6Al-4V [45]. The martensite phase is typical of as-built SLM parts, due to the high temperature gradient induced during the process.

**Figure 12:**
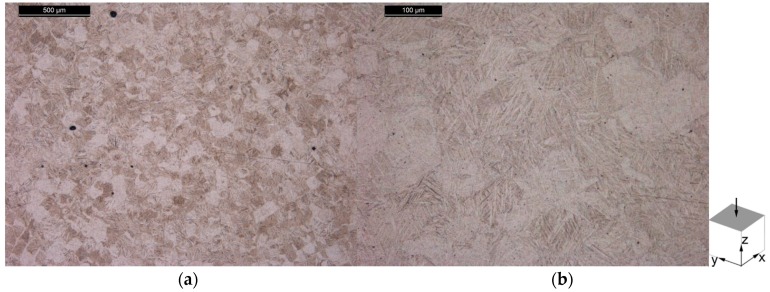
Microstructure of the horizontal plane of Batch 2 Ti-6Al-4V tensile specimen, in the stress relieve annealed condition, showing (**a**) an overview; and (**b**) microstructural features in more detail.

**Figure 13:**
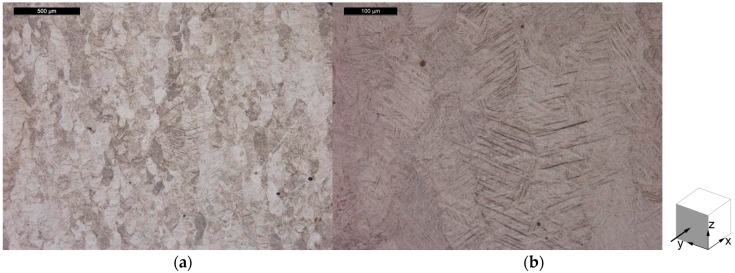
Microstructure of the vertical plane of Batch 2 Ti-6Al-4V tensile specimen, in the stress relieve annealed condition, showing (**a**) an overview; and (**b**) microstructural features in more detail.

The mean hardness value measured on the horizontal plane of the contaminated Batch 1 specimens is 397 HV ± 8 (SD). The mean value measured on the vertical plane is 392 HV ± 10 (SD). Concerning the non-contaminated Batch 2 specimens, a mean hardness value of 393 HV ± 7 (SD) was obtained for both planes. The difference between horizontal and vertical planes, as well as between contaminated and non-contaminated specimens was not significant and the values measured are in agreement with the values reported in previous studies [11]. Based on the small laser spot size of about 80 µm, the rapid scanning speeds applied in the AM process, the measured hardness values and the continuous cooling transformation diagram (CCT-diagram) for Ti-6Al-4V [46], it is concluded that the microstructure observed in Figure 12 and Figure 13 is fully martensitic with an acicular morphology.

The microstructure of the specimens from Batch 2 is shown Figure 12 for the horizontal plane and in Figure 13 for the vertical plane. With the exception of the tungsten particles, the microstructure does not differ between the contaminated Batch 1 specimens and the non-contaminated Batch 2 specimens. This observation indicates that the W particle contamination did not affect the microstructural evolution during the manufacturing process, and the subsequent heat treatment.

### 3.4. Powder Characterization

ASTM F3049 [47] defines feedstock characteristics which are considered useful to assure the confidence in the selected powder and in the final part properties, for powder-based AM processes. Among those characteristics are the particle size and its distribution, the morphology, the chemical composition, the flow characteristics and the density of the powder. Following these guidelines, the properties of the Ti-6Al-4V powder used to manufacture the first batch of specimens were assessed. 

**Figure 14:**
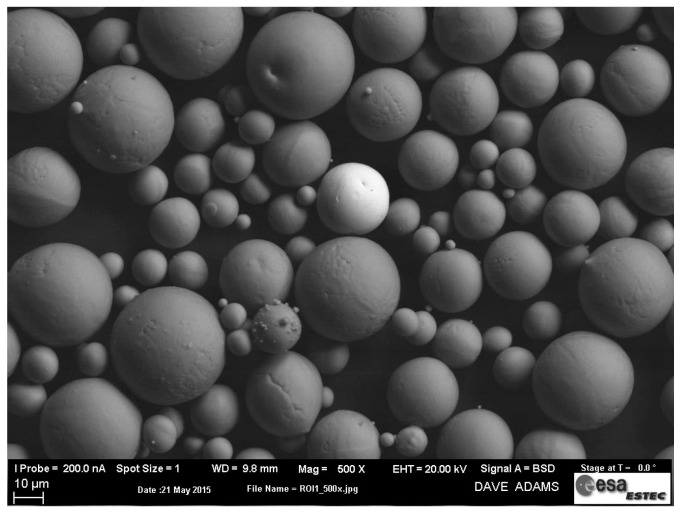
Back-scattered SEM image of Batch 1 powder, highlighting the sphericity of the particles.

The particle size distribution by volume was determined by Laser Light Scattering, and characterised as D_10_ = 20.2 µm, D_50_ = 31.8 µm and D_90_ = 51.4 µm. These values are comparable to what is reported in literature for Ti-6Al-4V powder [6,45]. The flowability was determined using the Hall flowmeter funnel, according to ASTM B213 [24]. The Ti-6Al-4V powder presented a flow rate of 25.4 s, which is consistent with the values available for commercial powders [15]. The morphology of the powder, as observed by SEM is shown in Figure 14 and is characterised by a high sphericity, with few satellite particles.

**Figure 15:**
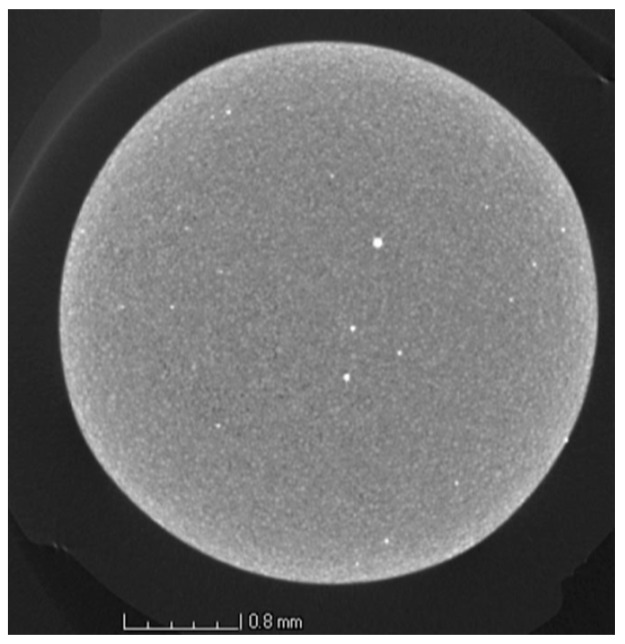
X-ray CT slice image of the powder from Batch 1, showing high density inclusions, visible as bright particles.

Similarly to the analysis performed on the tensile specimens, see Figure 7, X-ray CT was also used to characterise the defect population, namely inclusions, on the powder from Batch 1. Figure 15 reveals the presence of inclusions with a higher density than the Ti-6Al-4V base powder. They are visible as bright particles in the X-ray CT image. This method has been used by different authors as a characterization technique for powder [48,49], namely for size distribution and packing assessment. However, this technique has not been used to detect the presence of contamination on powder feedstock for Additive Manufacturing. This study shows that X-ray CT can be used for this purpose as a complementary technique, allowing the characterization of larger powder samples, when compared with SEM.

Table 3 show the results of the chemical composition analysis of the powder feedstock and AM samples, as well as the comparison with the values recommend in ASTM F2924 [25]. The chemical composition of the samples was in accordance with ASTM F2924 [25], for all elements. In particular, the presence of tungsten was not detected by the ICP-OES method. This could be explained by the fact that the tested sample was too small and W particles were not present during the analysis. Another justification could be related to the limitations of the ICP-OES method, namely the total dissolution of a given element, as it only measures liquid samples, and the interference between different elements [50].

### 3.5. Contamination Investigation

Understanding the origin of the observed high density brittle inclusions in the AM-processed specimens is of interest as it will allow avoiding similar contaminations in future AM builds. Contamination of the feedstock is the most likely source of inclusions in the final AM samples, as metal powder contamination is difficult to prevent. The Plasma Rotating Electrode Process (PREP), used for the production of the metal powders, was considered as the first potential source of contamination. It has been reported that the tungsten electrode used in the process can wear out and may lead to powder material containing tungsten particles [51,52]. Cross-contamination in the AM equipment was considered as a second potential source of contamination. After discussions with the supplier, cross-contamination was confirmed to be the source of the specimen contamination, as a batch of tungsten specimens was manufactured on the same AM machine, prior to the contaminated Ti-6Al-4V specimen batch.

Beyond the impacts on the mechanical properties illustrated earlier in this article—in particular the scattering of the obtained values—contamination in AM parts presents a threat for the ability of the parts to meet the requirements for Space applications. The case of tungsten inclusions is a known problem in Gas Tungsten Arc Welding where they are often considered as a source of failure of welded parts [53]. Other properties might be affected as well, such as corrosion resistance or stress corrosion cracking.

## 4. Conclusions

The mechanical properties of Ti-6Al-4V specimens manufactured by AM were assessed. The results from tensile testing showed values of strength above the minimum requirements of ASTM F2924 [25] for all specimen orientations in the build platform. However in the case of the vertical specimens built in the **Z** orientation, the yield strength and tensile strength results showed a larger scatter than in other orientations and the fracture elongation values were significantly lower than the requirements for AM parts.

From the fracture surface analysis, it was possible to detect the presence of particles of W in the tested specimens built in the **Z** orientation. The W particle contamination was also confirmed via X-ray CT, for both the **X** and **Z** orientations. The origin of this contamination was investigated and confirmed to be related to the cross contamination of the powder feedstock in the AM machine.

The microstructural study showed the presence of a fully martensitic microstructure with an acicular morphology, commonly observed for additive manufactured Ti-6Al-4V specimens.

The powder used was characterised using several techniques. Its morphology, size and size distribution is similar to what is reported in literature, despite of the contamination.

As cross contamination is considered to be a critical issue for AM Space hardware, different characterization methods were used to assess the chemical composition of the powder used. The methods most commonly used in the certification of powders for AM proved to be insufficient for the detection of the cross contamination. The W particles could only be detected by X-ray CT and their composition was confirmed by SEM, coupled with EDX.

Although the relation between the presence and the distribution of W particles and the tensile properties could not be fully characterised—in particular with respect to the anisotropy of the elongation between various specimen orientations—the negative impact of the W contamination on the fracture elongation and on the repeatability of the test results could be clearly observed. A possible explanation was given by the analysis of the size distribution of the inclusions in the contaminated samples, through the X-ray CT data. This indicated that the vertical samples (**Z** direction) show a larger number of inclusions, where the area occupied by these particles presents a larger variability than for the horizontal samples. Hence, a reduction of the effective load-bearing area for the tensile tests is assumed, and the inclusions are considered to be a stress concentration factor. Both aspects would contribute to the impairing of the mechanical properties and may justify the results obtained.

In addition, cross contamination of the feedstock is expected to have a negative impact on other properties of the AM material, such as Stress Corrosion Cracking and corrosion resistance. The detection of foreign particles in the feedstock was demonstrated to be challenging. As such, the use of only one powder type per AM equipment is recommended for the manufacture of parts in critical applications, such as space hardware.

## Figures and Tables

**Table 1:** Tensile testing results (average ± standard deviation, SD) for specimens from Ti-6Al-4V Batch 1 in the stress relief annealed state (670 °C, 5 h).

Specimen Orientation	Yield Strength [MPa] ± SD	Tensile Strength [MPa] ± SD	Fracture Elongation [%] ± SD
**X**	1092 ± 12	1168 ± 12	7.5 ± 1
**111**	1134 ± 6	1192 ± 6	8.3 ± 0.7
**Z**	1100 ± 56	1186 ± 45	2.9 ± 0.5

**Table 2:** Statistical variables defining the distributions of inclusions projection area, in the yz, xz and xy planes, for samples built in **X** and **Z** directions. All units are in mm^2^.

Build Direction	No. Total	Plane	Total Area	Minimum	1st Quartile	Median	3rd Quartile	Maximum
**X**	153	P_yz_	1.78 × 10^−1^	1.90 × 10^−4^	1.06 × 10^−3^	1.15 × 10^−3^	1.28 × 10^−3^	3.23 × 10^−3^
		P_xz_	1.83 × 10^−1^	1.90 × 10^−4^	1.06 × 10^−3^	1.18 × 10^−3^	1.34 × 10^−3^	3.07 × 10^−3^
		**P_xy_**	**1.85 × 10^−1^**	**1.60 × 10^−4^**	**1.06 × 10^−3^**	**1.17 × 10^−3^**	**1.30 × 10^−3^**	**3.01 × 10^−3^**
**Z**	210	P_yz_	2.23 × 10^−1^	1.90 × 10^−4^	8.3 × 10^−4^	1.02 × 10^−3^	1.22 × 10^−3^	3.20 × 10^−3^
		P_xz_	2.43 × 10^−1^	1.90 × 10^−4^	9.4 × 10^−4^	1.09 × 10^−3^	1.28 × 10^−3^	4.48 × 10^−3^
		**P_xy_**	**2.92 × 10^−1^**	**1.90 × 10^−4^**	**1.02 × 10^−3^**	**1.34 × 10^−3^**	**1.60 × 10^−3^**	**4.55 × 10^−3^**

**Table 3:** Chemical composition of used feedstock and AM-processed material obtained from powder Batches 1 and 2.

Element [wt %]	H	O	N	C	Fe	Al	V	W	Ti
Method	CGHE	CGHE	CGHE	Combustion method	Flame AAS	ICP-OES	ICP-OES	ICP-OES	ICP-OES
Powder sample (Batch 1)	0.008	0.2	0.014	0.012	0.19	6.69	4.06	<0.005	Balance
Bulk sample (Batch 1)	0.004	0.21	0.016	0.012	0.18	6.444	3.99	<0.005	Balance
Bulk sample (Batch 2)	0.003	0.18	0.022	0.008	0.19	6.33	3.91	<0.005	Balance
*ASTM F2924*	*0.015*	*0.20*	*0.05*	*0.08*	*0.30*	*5.50–6.75*	*3.50–4.50*	*0.10*	*Balance*
